# Freiburg Neuropathology Case Conference: A 6-year-old Girl Presenting with Vomiting and Right-sided Facial Paresis

**DOI:** 10.1007/s00062-021-01069-3

**Published:** 2021-09-01

**Authors:** U. Taschner, M. Diebold, M. J. Shah, M. Prinz, H. Urbach, D. Erny, C. A. Taschner

**Affiliations:** 1 Department of Neuroradiology, Medical Centre—University of Freiburg, Breisacherstraße 64, 79106 Freiburg, Germany; 2 Department of Neuropathology, Medical Centre—University of Freiburg, Freiburg, Germany; 3 Department of Neurosurgery, Medical Centre—University of Freiburg, Freiburg, Germany; 4 Faculty of Medicine, University of Freiburg, Freiburg, Germany

**Keywords:** Pilocytic astrocytoma, Supratentorial ependymoma, Atypical teratoid/rhabdoid tumor, Choroid plexus carcinoma, Primary central nervous system neuroblastoma

## Case Report

**Fig. 1:**
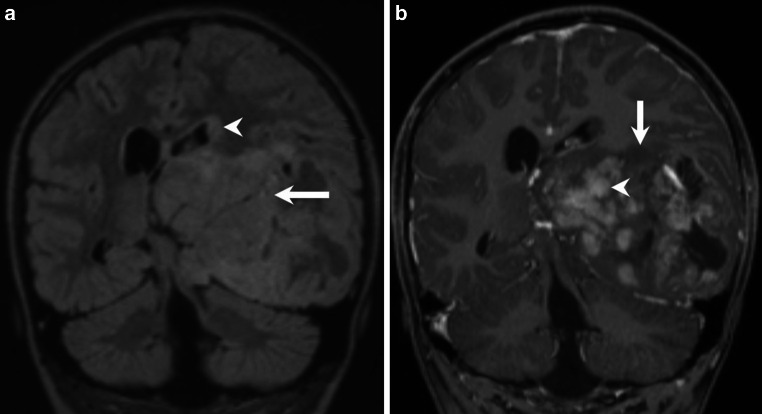
Coronal fluid-attenuated inversion recovery (FLAIR) images (**a**) show a huge space-occupying lesion of the left temporal lobe (*arrow*) causing a distinct midline shift to the right. A subependymal hypersignal can be seen at the level of the cella media of the left lateral ventricle (*arrowhead*). On coronal T1 weighted images after administration of gadolinium (**b**) the lesions shows a heterogeneous pattern of enhancement with a large number of well-delineated portions showing homogeneous enhancement (*arrowhead*) and areas with virtually no contrast enhancement (*arrow*)

**Fig. 2:**
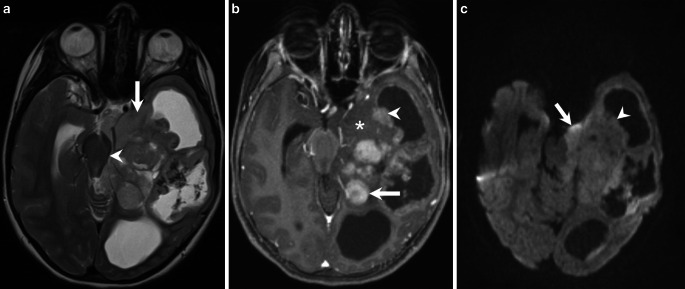
Axial T2 weighted image (**a**) shows the lesion affecting the mesial temporal lobe (*arrow*) as well as the parahippocampal gyrus. In addition, subependymal spread at the level of the temporal horn of the left lateral ventricle is visible. Exophytic parts of the tumor expand into the subarachnoid space causing massive compression and dislocation of the midbrain (*arrowhead*). Axial T1 weighted images after administration of contrast agent (**b**) show areas of homogeneous tumor enhancement at the parahippocampal gyrus (*arrow*) as well as subependymal tumor portions of the temporal horn (*arrowhead*). Note that the mesiotemporal tumor parts as well as the exophytic tumor portions extending into the ambient cistern do not show any contrast enhancement (*asterisk*). On diffusion weighted images (b-value: 1000, **c**) contrast enhancing (*arrowhead*) as well as non-enhancing parts of the tumor (*arrow*) clearly show restricted diffusion indicative of a hypercellular tumor

**Fig. 3:**
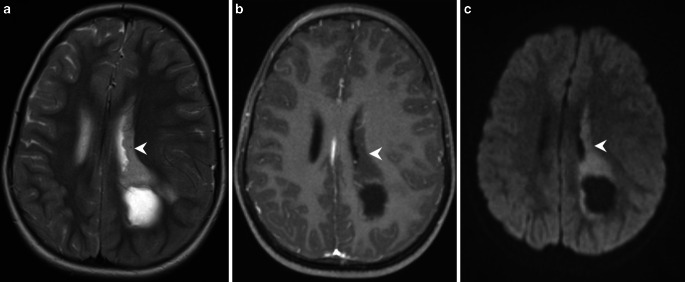
Axial T2 images (**a**) show subependymal tumor portions at the level of the cella media (*arrowhead*). On T1 weighted post-contrast images (**b**) this part of the tumor does not show any enhancement (*arrowhead*). On diffusion weighted images (b-value: 1000, **c**) the diffusibility within the tumor is clearly restricted (*arrowhead*)

A 6-year-old girl presented with recurrent daily morning vomiting starting 1 week ago. The clinical examination showed a mild right-sided facial paresis and bilateral congestive papillae. Magnetic resonance imaging (MRI) of the brain showed an extensive heterogeneous temporoparietal tumor formation of the left side with intraventricular infiltration (Figs. 1, 2 and 3). Urgent surgical decompression was indicated. Temporofrontal craniotomy was performed with the patient under general anesthesia in the supine position under neuromonitoring. The brain stem was decompressed by partial temporal resection of the tumor. The tumor appeared soft, bled heavily upon resection and was difficult to separate from the surrounding brain tissue, finally we only performed a partial resection.

After surgery, the child promptly woke up and was discharged home without focal neurological deficit on postoperative day 7. Postoperative computed tomography (CT) revealed the extent of the tumor resection, with tumor remnants located in the midline, along the lateral ventricles and in the course of the fornix and septum pellucidum (not shown). An MRI control 4 weeks later showed tumor progression (not shown). After discussion of the case in our multidisciplinary tumor board, chemotherapy with carboplatin-etoposide was initiated.

## Imaging

The MRI of the brain revealed a massive space-occupying lesion affecting the mesial temporal lobe, and the underlying white matter of the temporal lobe, the parahippocampal gyrus, as well as the left thalamus. In addition, subependymal growth of the lesion expanding into the ventricular system and subsequent ventricular distension is present at the level of the left temporal horn of the lateral ventricle, the occipital horn of the left lateral ventricle, and the cella media of the left lateral ventricle. Exophytic tumor expansion into the subarachnoidal space causing marked compression of midbrain structures are present at the level of the mesial temporal lobe and the parahippocampal gyrus (Figs. 1, 2 and 3). On fluid attenuated inversion recovery (FLAIR) images (Fig. 1a), and T2 weighted images (Fig. 2a and 3a) the lesions appear relatively homogeneously hyperintense. In addition, cystic components are present indicating regressive changes of the tumor matrix. On T1 weighted images obtained after administration of gadolinium the lesion shows a heterogeneous pattern of enhancement (Fig. 1b, 2b and 3b). The lesion presents with a large number of well-delineated portions showing homogeneous enhancement at the level of the thalamus (Fig. 1b), the parahippocampal gyrus, and subependymal tumor growth along the temporal horn (Fig. 2b). On the other hand, there are tumor portions that show no enhancement of contrast. These include the mesiotemporal as well the exophytic tumor portions extending into the subarachnoidal space (Fig. 1b), but also subependymal tumor portions of the cella media (Fig. 1c). On diffusion-weighted images (b-value: 1000) the tumor shows restricted diffusion suggesting a hypercellular tumor (Fig. 2c and 3c). The diffusion restriction is unrelated to the variable degree of contrast enhancement. On CT images obtained immediately after tumor resection the remaining tumor parts did not show any apparent calcification (not shown).

## Differential Diagnosis

### Pilocytic Astrocytoma

Pilocytic astrocytoma (PA) is the most common primary brain tumor of childhood accounting for 15% of all pediatric brain tumors with a peak incidence between 5 and 15 years. Pilocytic astrocytomas are low grade World Health Organization (WHO) type I tumors showing a strong association with neurofibromatosis type 1 (NF1) without any gender predisposition [1]. Pilocytic astrocytomas typically arise from midline structures. The most common location in non-NF1 patients is the cerebellum in 60%, whereas PA of the optic pathway are particularly common in NF1 patients. Less common locations include the hypothalamus, brainstem and cerebral hemispheres, cerebral ventricles, velum interpositum and the spinal cord [2].

Pilocytic astrocytoma have a wide range of imaging appearances. On MR imaging four predominant imaging patterns have been described: (a) mass with a nonenhancing cyst and an intensely enhancing mural nodule (21% of cases), (b) mass with an enhancing cyst wall and an intensely enhancing mural nodule (46%), (c) necrotic mass with a central nonenhancing zone (16%), and (d) predominantly solid mass with minimal to no cyst-like component (17%). Calcifications are present in 20% of cases [3]. Given the localization and the extent of the lesion pilocytic astrocytoma was considered a less likely differential diagnosis.

### Supratentorial Ependymoma

Ependymoma is the number three of the most common intracranial neoplasm in children. They arise from ependymal cells of the central nervous system (CNS) and can occur anywhere within the CNS [4]. The incidence is bimodal with one peak between 1–5 years and a smaller peak during the 2–3 decade of life with a male predisposition. Most common symptoms are seizures, focal motor or sensory deficits and headache, but also when large, signs of raised intracranial pressure and obstructive hydrocephalus especially in infratentorial location may occur. Supratentorial location accounts for only one third of ependymomas. They typically arise from the ependymal lining of the ventricular system or fetal ependymal rests. The majority of supratentorial ependymomas are located within the white matter or cortex of the frontal lobe. Ventricular localization is less common. In the WHO classification from 2021, supratentorial ependymomas are classified based on molecular features into two subgroups. RELA fusion-positive supratentorial ependymomas seem to affect older children and have a poor prognosis, whereas supratentorial ependymomas displaying YAP‑1 fusions affect children below 3 years of age [5, 6].

At MRI ependymomas are isointense to slightly hypointense on T1-weighted images and isointense to hyperintense on T2 weighted images. Heterogeneous enhancement is seen on contrast-enhanced images. Restricted diffusion is regularly present, most likely reflecting high cellularity [7]. On CT images, the soft tissue component is commonly hypoattenuating to isoattenuating. Ependymomas frequently demonstrate cystic components, and areas of small chunky calcification. Occasionally intratumoral hemorrhage may be present [7]. Because of ependymal location of the lesion, as well as the diffusion restriction, supratentorial anaplastic ependymoma was considered a valid differential diagnosis in our case.

### Atypical Teratoid/Rhabdoid Tumor (AT/RT)

Atypical teratoid/rhabdoid tumors (AT/RT) are rare, WHO grade IV tumors of the CNS. In > 80% of cases children less than 3 years of age are affected. There is no gender predisposition. They are among the fastest growing CNS tumors with a short interval to symptom onset. By the time of diagnosis, they often reach sizes > 3 cm. Most common symptoms are rapidly increasing lethargy, vomiting and increased head circumference. Smaller supratentorial lesions may cause seizures [8]. The AT/RT may occur supratentorially or infratentorially. Infratentorially they appear off-midline and may resemble medulloblastomas, 12% of AT/RT are found bihemispherically and 15–20% are disseminated at the time of initial diagnosis. Histologically, AT/RTs are embryonal tumors that contain a rhabdoid component as well as areas with primitive neuroectodermal, mesenchymal, and epithelial features. The current WHO classification of 2021 places AT/RT under embryonal tumors. This group of tumors includes medulloblastoma, atypical teratoid/rhabdoid tumor, embryonal tumor with multilayered rosettes, C19MC-altered and embryonal tumor with multilayered rosettes, pineoblastoma, pituitary blastoma, CNS neuroblastoma, and ganglioneuroblastoma [9]. Diagnosis of AT/RT requires confirmation of specific genetic aberration, such as loss of INI1 tumor suppressor gene on chromosome 22 or BRG1 gene [10].

The use of CT imaging often shows a hyperattenuated mass lesion commonly containing cystic structures and/or hemorrhage. Some tumors contain fine calcifications and signs of obstructive hydrocephalus may be present [11, 12]. On T2-weighted images, AT/RTs appear heterogeneous with hyperintense cystic foci, which are hyperintense on T1-weighted images. In addition, restricted diffusion may be present in solid parts of the tumor. Contrast enhancement on T1 weighted images is mostly heterogeneous and can be nodular [13]. Considering the patient’s age and the imaging findings, AT/RT seemed to be a likely differential diagnosis.

### Choroid Plexus Carcinoma

Choroid plexus carcinomas (CPC) are rare entities. They are rapidly growing CNS neoplasms, originating from the epithelium of the choroid plexus and are classified as WHO grade III tumors. They occur predominantly in children, typically younger than 5 years old, most of them (70%) are affected before the age of 2 years with male predisposition [14]. Choroid plexus carcinoma typically occurs in the ventricular system with the lateral (50%) and the 4th ventricle (40%) being the most common locations. Extraventricular manifestation of CPC has been reported in the literature [15]. Clinically CPC presents with unspecific symptoms, such as nausea, vomiting, headache, obtundation as well as focal neurologic deficits.

On MR imaging CPC present on T1-weighted and T2-weighted images as heterogeneous isointense to hypointense intraventricular mass lesions with lobulated or irregularly marginated, papillary appearance. In addition, degenerative changes with cystic transformation, necrosis, and hemorrhage can regularly be found. On diffusion-weighted images CPC can show restricted diffusion, a finding correlated with a poor prognosis [16]. Because of its relation to the ventricular system, CPC was considered a likely diagnosis in the present case.

### Primary Central Nervous System Neuroblastoma (PCNSN)

Primary central nervous system neuroblastoma (PCNSN) is a newly defined intracranial embryonal WHO grade IV tumor. These rare tumors have a primitive neuronal architecture and occasional neurocytic differentiation. They mainly affect children under 5 years of age with a female prevalence. The PCNSNs are in most cases located supratentorially and present with a wide spectrum of symptoms due to their variety of possible locations and sizes. Infants may present with few or no signs or symptoms, even when the tumor has reached a considerable size. This is most likely due to compensatory and adaption mechanisms of the surrounding brain structures [17]. In most cases, PCNSN appear as heterogeneous mass lesions with solid and cystic components. The solid tumor parts appear hypointense to isointense on T1-weighted images, and hyperintense on T2-weighted images. Perifocal edema is inconsistent, whereas vascular structures crossing the tumor are not uncommon. PCNSN show markedly heterogeneous enhancement of gadolinium. On diffusion-weighted images the tumor often shows restricted diffusion, whereas the relative cerebral blood volume is reportedly elevated [17]. Although PCNSN are rare entities, we included this tumor in our differential diagnoses.

## Histology and Immunohistochemistry

**Fig. 4:**
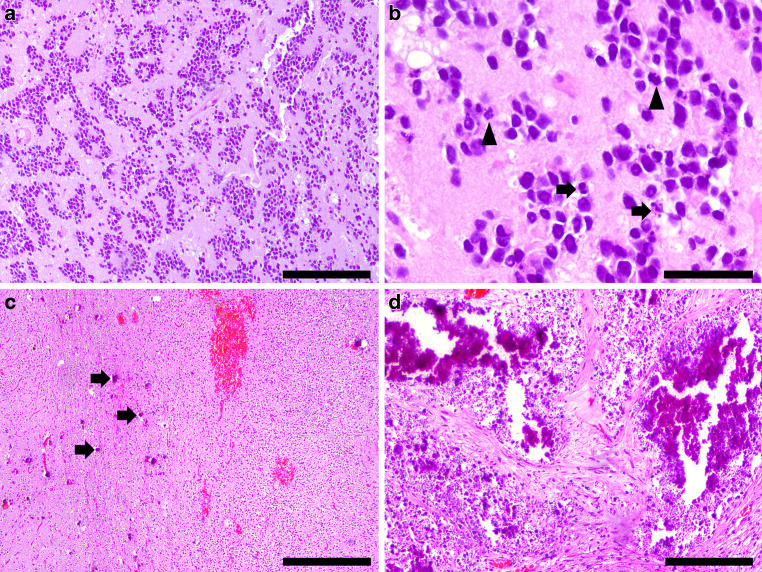
Hematoxylin-eosin stained sections with characteristic features of a CNS neuroblastoma (**a**–**d**). Intraoperative specimen (**a**) showing a dense and pleomorphic neoplasm with small to maximally medium sized, mainly round nuclei surrounded by neuropil-like matrix. Size bar = 200 µm. Intraoperative specimen (**b**) depicting the aspects of numerous mitotic figures (*arrowheads*) and apoptotic cells (arrows). Size bar = 50 µm. Biopsy material (**c**) with areas of different cell density, as well as sporadic small calcifications (*arrows*). Size bar = 200 µm. Section (**d**) showing regions with large granular calcifications. Size bar = 200 µm

The initial hematoxylin and eosin (H&E) stained section of the formaldehyde-fixed and paraffin-embedded intraoperative biopsy material revealed a dense and pleomorphic neoplasm with small to maximally medium sized, mainly round nuclei. Regionally, tumor cells clustered in groups surrounded by a neuropil-like matrix (Fig. 4a). Brisk mitotic activity and apoptotic cells (Fig. 4b) suggested a malignant entity. Complementing biopsy material further enriched the palette of morphological features of this tumor, ranging from densely packed areas characterized by small, round, blue cells with sparse cytoplasm and an occasional palisading pattern, to regions with moderate cell density and a fibrillar matrix (Fig. 4c). In the latter regions, sporadic small calcifications (Fig. 4c, arrowheads) attributed to an oligoid impression. Only relatively small fractions within the tumor tissue were necrotic, infrequently collocated with larger, granular calcifications (Fig. 4d). Tumor growth appeared to partially follow meningeal structures and diffusely infiltrate the surrounding parenchyma.

**Fig. 5:**
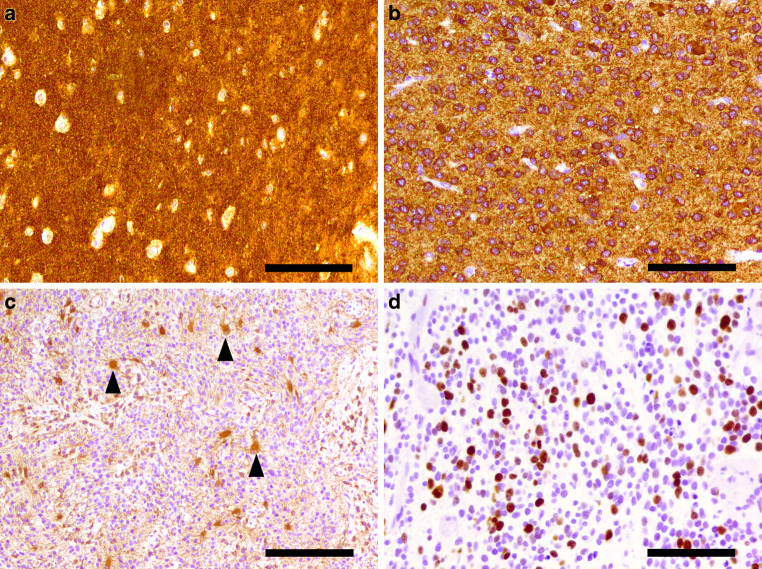
Immunohistochemical analysis of this CNS neuroblastoma case (**a**–**d**). Staining of the synaptic vesicle protein synaptophysin (**a**) revealing abundant expression in tumor cells. Size bar = 100 µm. Immunohistochemical staining of the neural antigen MAP2 (**b**) showing strong expression in tumor cells. Size bar = 100 µm. Immunohistochemical staining of the glial fibrillary acid protein (GFAP, **c**), limited to distinct regions within the tumor, showing moderate astrogliosis (exemplary astrocytes marked by *arrowheads*). Size bar = 200 µm. Immunohistochemical staining with the proliferation marker Ki67/MIB1 (**d**) showing high frequency of proliferating tumor cells. Size bar = 100 µm

Immunohistochemistry with antibodies against the synaptic vesicle protein synaptophysin (Fig. 5a) and against the neural antigen MAP2 (Fig. 5b) showed strong positive reactions in tumor areas of both high and medium cell density. While the oligodendrocyte transcription factor OLIG2 was expressed in a subset of tumor cells, the glial fibrillary acid protein (GFAP) was limited to relatively few cells showing only local moderate astrogliosis (Fig. 5c). The tumor cells showed a negative reaction for vimentin and CD99, a marker typical for Ewing’s sarcoma. The nuclear expressions of the transcriptional regulator ATP-dependent X‑linked helicase (ATRX) and the tumor suppressor gene integrase interactor 1 (INI) were maintained in the tumor cells. Immunohistochemistry with a mutation-specific (R132H) antibody against isocitrate dehydrogenase 1 (IDH1) and mutation-specific (G34V, G34R, K27M) antibodies against histone 3‑point mutations revealed no specific reaction in the tumor cells. The expression of CD34 was limited to vascular endothelial cells. Staining against epithelial membrane antigen (EMA) did not reveal dot-like or ring-like structures in any tumor cells. Along with numerous hyperchromatic nuclei, mitotic figures were a frequent characteristic in small round cells and the proliferation index was increased. In Ki67/MIB1 immunohistochemical staining, a total of approximately 20%, locally—mostly in dense areas—even up to 40–50% of tumor cells were stained (Fig. 5d). In summary, the histomorphological appearance of a poorly differentiated tumor with high proliferation rate and immunohistochemical characterization of neuroectodermal origin categorize this case as an embryonal tumor of the CNS. The co-existence of dense areas of small, round, blue embryonal cells and areas of pleomorphic, neurocytic cells in a neuropil-rich stroma leads to the diagnosis of a central nervous system (CNS) neuroblastoma (WHO grade IV). Key features of this entity visible in this case include co-expression of synaptophysin and OLIG2 and necrotic regions with granular calcifications [18, 19]. In addition, a molecular analysis of the biopsy tissue assessing the tumor cell DNA methylation profile was performed. The identified copy number variation profile revealed increases on chromosomes 1q, 3q and 17q as well as losses on chromosomes 3p and 6q. To discover common patterns with a library of established tumor profiles, the results of this methylation analysis were assessed using the Heidelberg molecular neuropathology classifier version v11b4 [20]. This classifier showed the highest accordance with the methylation class of CNS neuroblastomas with forkhead box R2 (FOXR2) activation (calibrated score 0.98). Assessment by the brain tumor reference center in Bonn, Germany, independently confirmed the diagnosis.

Initial differential diagnostic considerations of the tumor at hand comprised other CNS embryonal tumors, like CNS ganglioneuroblastoma, medulloepithelioma, and ependymoblastoma (all WHO grade IV), and mixed neuronal-glial tumors like the diffuse leptomeningeal glioneural tumor [21]. A malignant glioma was also originally considered, but overruled by the relative sparsity of glial marker expression. In comparison to mixed neuronal-glial tumors, the high proliferation rate, abundance of primitive cells and preponderance of neural markers favored a CNS embryonal tumor. Among embryonal tumors, the above described histological key features in combination with the absence of ganglion cells (ganglioneuroblastoma), pseudostratified neuroepithelial structures (medulloepithelioma) and prominent rosettes (ependymoblastoma) led to the diagnosis of a CNS neuroblastoma (WHO grade IV).

## Diagnosis

### Central Nervous System (CNS) Neuroblastoma (WHO-grade IV)

Historically, this former group of so-called primitive neuroectodermal tumors (PNETs) of the CNS originated from an concept analogous to the related and more frequent medulloblastomas the PNET of the cerebellum [22]. They were first subdivided into four distinct groups in the 2007 WHO classification of CNS tumors, based on histomorphological criteria [23]. In up to 61% of cases, these histologically classified CNS-PNETs could be reclassified to established reference CNS tumors (e.g. high-grade gliomas), based on their molecular profiles [22]. Sturm et al. assessed the remaining tumors with a genuinely different genetic signature, clustering them into four new entities. Among these, CNS neuroblastoma with FOXR2 activation shares the methylation profile and histological features of the tumor described here. Recently, the cIMPACT-NOW consortium suggested that this new entity be integrated in the next WHO classification of CNS tumors [18].

A CNS neuroblastoma is a rare entity. Several small case series suggest a mean age of 5–9 years and a female:male ratio of approximately 2:1 [17, 24, 25]. Detailed epidemiological information is limited due to the low frequency and relatively new and evolving diagnostic criteria of CNS neuroblastomas, specifically with respect to associated mutations. Besides the frequent FOXR2 activation, singular cases may also be characterized by focal MYC amplification [22]. In comparison to other CNS embryonal tumors, a slightly less unfavorable prognosis has been proposed for this entity [17]. Yet the prognostic value of either of these alterations (FOXR2, MYC) in CNS neuroblastomas remains to be determined [18]. Overall, the diagnosis of these heterogeneous and poorly differentiated CNS embryonal tumors remains challenging based on histomorphological features alone and profited from recent advances in molecular pathological assessment.
